# The Investigation of reasons for differences between the National Reports and World Health Organization estimates about road traffic deaths

**Published:** 2019-08

**Authors:** Alireza Razzaghi, Hamid Soori, Ardeshir Khosravi, Amir Kavousi Dolanghar, Alireza Abadi

**Affiliations:** ^*a*^Safety Promotion and Injury Prevention Research Center, Shahid Beheshti University of Medical Sciences, Tehran, Iran.; ^*b*^Department of Statistics and Informatics, Iranian Ministry of Health and Medical Education, Tehran, Iran.; ^*c*^School of Health, Safety and Environment, Shahid Beheshti University of Medical Sciences, Tehran, Iran.; ^*d*^Department of Biostatistics, Shahid Beheshti University of Medical Sciences, Tehran, Iran.

## Abstract

**Background:**

Road traffic injuries (RTIs) and its related deaths is one of the main public health problems. The national and international efforts for reducing the RTIs and RTDs can only be successful if there be validated RTIs registry systems or precis related estimations. This information is important not only in the national but also at the international level because of carrying out the national and international coordinated efforts. In World Health Organization (WHO) reports in the years of 2009, 2011, 2013, 2015 and 2017 there are some differences about the numbers of RTDs between the national reports and WHO estimates for some high, middle and low-income countries. This study was aimed to investigate the differences in the numbers of RTDs between the national reports and WHO estimates.

**Methods:**

This is a combined study of review and systematic review. Review study was done on the WHO reports in the years of 2009, 2011, 2013, 2015 and 2017. Also, a systematic review has done on original research during the January 2008 and 2018 using the databases of EMBASE, CINAHL, PubMed, Google Scholar, Medline and ISI Web of Science. The related keywords determined for implementing the search.

**Results:**

The findings of this study show that there are some differences between the national reports and WHO estimates about the RTDs. The reasons for these differences are related to the following issues; 1) Road traffic registry system (RTRS) and its related issues. The quality of RTRS is different among the high, middle and low-income countries. 2) The issues related to modeling method which was done by WHO such as epidemiological differences among countries which were not considered in WHO modeling.

**Conclusions:**

Validated information about RTDs is very important to planning and implementing the effective programs for prevention and reducing the RTIs and RTDs. Given to observed differences between the countries national reports and WHO estimates and its related reasons, there is a need to pay more attention to considering in reports and planning.

**Keywords:**

Deaths, Road traffic crashes, National reports, Estimation, World Health Organization

